# Placebo and Side Effects Confound Clinical Trials on New Antitussives

**DOI:** 10.1007/s00408-021-00458-2

**Published:** 2021-07-19

**Authors:** Ronald Eccles

**Affiliations:** Cardiff School of Biosciences, Cardiff University, Museum Avenue, Cardiff, CF10 3AX UK

**Keywords:** Cough, Placebo effect, Additivity, Blinding, ATP antagonist, Side effects

## Abstract

This review discusses how the placebo effect related to treatment side effects may confound clinical trials on antitussives and specifically looks at the implications for trials on ATP antagonists. These new antitussives have distinctive side effects on the sensation of taste, and investigators have expressed concerns that this may unblind the clinical trials. Blinding is an essential component of trial design, but the degree of blinding in trials is rarely assessed. The assumptions of additivity and balance in clinical trials are discussed as important factors that allow assessment of the pharmacological activity of an antitussive. How side effects unbalance a clinical trial by amplifying the placebo effect of active treatments is discussed. The point is made that unblinding of trials invalidates any assessment of efficacy but that there is little interest or discussion about this fundamental aspect of trials. Proposals are discussed which may improve the blinding of trials and control placebo effects by changes to participant information, trial design, patient selection and use of active placebos. The issue of unblinding of clinical trials is not a new issue, but if real progress is to be made in developing new antitussives, then it is an issue that needs to be urgently addressed.

## Introduction

There is a great need for new antitussive medicines as “cough remains a serious unmet clinical problem” [1], but cough clinical trials are especially at risk from confounding placebo effects, as cough is under voluntary control and is related to a sensation of irritation in the airway and a ‘drive to cough’ [2–4]. The double-blind, randomised, placebo-controlled clinical trial design is the gold standard method of testing the efficacy of new antitussive medicines, and all the recent clinical trials on new antitussives use this trial design. A key aspect of the trial design is that both the patient and all those concerned with the conduct of the trial are blinded as to which patients are taking a placebo treatment or the active medicine under investigation. In order to blind the study, the placebo treatment and active treatment must be identical in all sensory aspects such as shape and colour of tablet and smell and this is usually acknowledged in the description of methods as the use of a “matched placebo”. However, it is not always possible to control the side effects of the active treatment such as sedation, nausea and disturbances of taste or any other side effect which can be sensed by the patient and which may be apparent to those involved in the conduct of the study. Side effects associated with the active treatment may unblind the study. The importance of side effects in unblinding clinical trials was clearly stated by Marini et al. (1976) [5] when discussing clinical trials on lithium, “The validity of the double-blind design is therefore in question, in principle, in every experiment employing it, and is especially vulnerable when an agent with pronounced or unique side effects is studied”. Side effects have also been reported to ‘amplify’ the placebo effect of a treatment and convince patients of the efficacy of a treatment [6].

The role of side effects as a confounding factor in the conduct of placebo-controlled clinical trials has received much attention in studies on psychoactive medicines such as lithium [5], tricyclic antidepressants [7] and also on trials for chronic pain [8], but there has been little interest in the significance of this issue in clinical trials for antitussives, despite the fact that many antitussive medicines have significant side effects that can be readily recognised by patients and those involved in conducting the clinical trials.

The issue of side effects as a factor that confounds the validity of clinical trials has recently become more important with studies on new antitussives such as those on ATP receptor antagonists where the test medicines have specific and readily identifiable side effects on the sensation of taste [9, 10]. This review will discuss the role of side effects of antitussives in clinical trials and how they may confound the interpretation of the results of these trials.

### The Assumption of Additivity and Balanced Clinical Trials

**Fig. 1:**
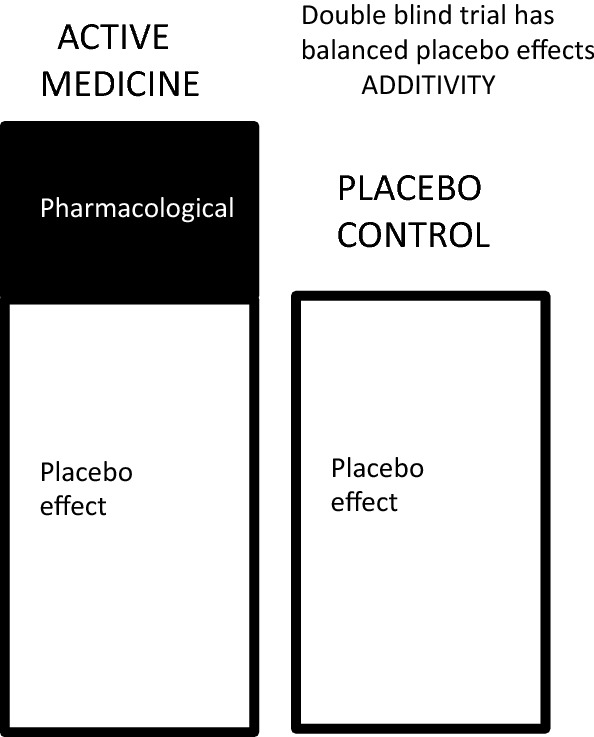
Efficacy of an antitussive medicine consists of a pharmacological effect and a placebo effect. The placebo effects of both arms of the study are balanced, and subtraction of the efficacy of the placebo from the efficacy of the active arm allows measurement of pharmacological antitussive efficacy. This is the principle of additivity in clinical trials

When considering the placebo response in placebo-controlled clinical trials, it is important to understand that the placebo arm of the trial is conducted in order to control for a placebo response in the treatment arm of the study. The rationale for using a placebo control is simply illustrated in Fig. 1. The figure illustrates that the antitussive activity of a cough medicine is determined by two components: a pharmacological response and a placebo response. In order to determine the magnitude of the pharmacological response, it is necessary to compare it with the antitussive efficacy of a matched placebo and to subtract the efficacy of the placebo arm from the active treatment arm.

There is an assumption of additivity in placebo-controlled trials that the placebo response in the placebo arm of the study exactly matches the placebo response in the active-medicine arm, and if this assumption is not correct, then it is not possible to determine the magnitude of any pharmacological response of the active medicine [11]. This is not a new issue, as it was raised in 1987 by Moscucci et al. in 1987 [12] and Fisher and Greenberg in 1993 said that there were major doubts about the validity of the double-blind placebo-controlled trial and inertia in scientific systems to acknowledge that there were serious challenges to the standard double-blind format for clinical trials [7].

### Factors Influencing the Magnitude of the Placebo Response in a Cough Medicine

The magnitude of the placebo response of a cough medicine or any other medicine is determined by the expectancy and belief of the patient that they are taking an active medicine [13]. If the medicine can be administered without the patient knowing it has been administered, i.e. no sensory impact by visual or other clues, then there is a much reduced response associated with an active treatment, as the placebo response is absent [14]. In contrast, many freely available over the counter cough medicines (OTC) enhance the sensory impact of the medicine by including sapid and sensory excipients such as cooling, warming and tingling agents that provide a powerful sensory impact to the patient and enhance the placebo response of the medicine [15].

In cough clinical trials, it could be argued that hard outcome measures such as cough counts are less susceptible to placebo response than subjective measures of cough but this is unlikely, as cough is mediated by a sensation of an “urge to cough” [3] and the magnitude of this sensation will be subject to a placebo response.

**Fig. 2:**
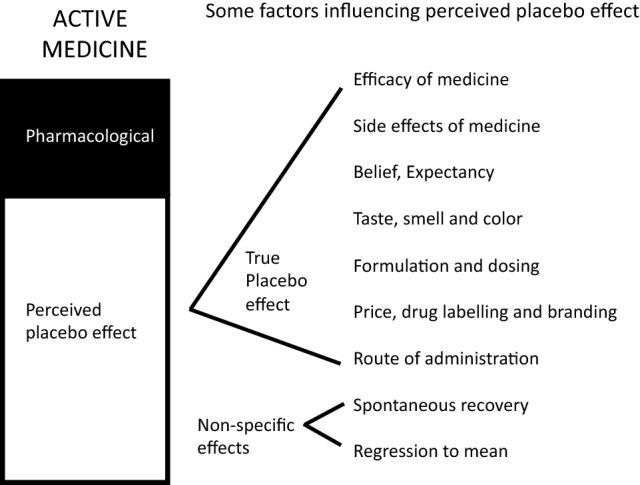
Factors influencing the magnitude of the placebo effect in a cough medicine. The perceived placebo effect of a medicine consists of a true placebo effect and non-specific effects. Note that the efficacy of the medicine and any side effects may contribute to the placebo effect of the active medicine and may also influence the placebo arm of the study if patients expect side effects

The factors influencing the magnitude of the placebo response are illustrated in Fig. 2 and these have been recently discussed by Meissner and Linde (2018) [16] and Eccles (2020) [17]. The placebo response can be considered as consisting of two components: a perceived placebo effect and a true placebo effect, as first defined by Ernst and Resch (1985) and applied to cough medicines by Eccles (2007) [18, 19]. For the purpose of this review, it is the side effects of a cough medicine that are of interest, as these influence the magnitude of the true placebo effect, and the various other factors that may influence the perceived placebo effect will not be discussed in detail.

### Do Side Effects Increase the Efficacy of the Active Treatment?

Medicines are administered to achieve a pharmacological response such as an antitussive or analgesic effect, but there are few medicines that achieve efficacy without side effects. Silent side effects such as minor changes in electrolytes or blood pressure may not influence the placebo response, as the patient does not sense any side effect of the medicine. But other side effects such as dry mouth, sedation and nausea will be sensed by the patient and these will confirm to the patient that they are receiving an active medicine, and this may unblind the study and enhance the efficacy of the medicine by increasing the true placebo effect of the medicine [20].

**Fig. 3:**
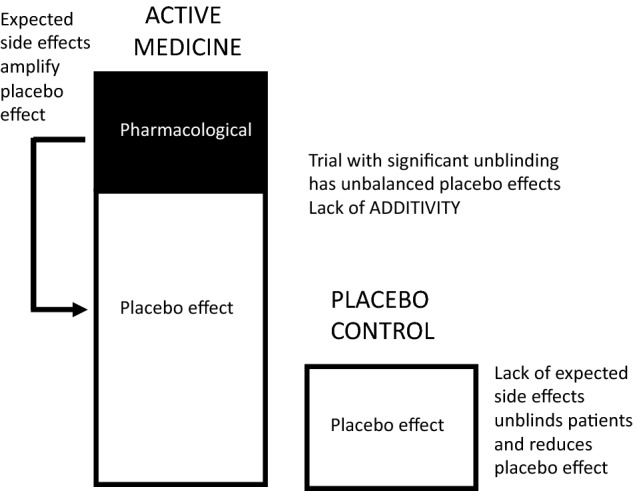
The active medicine has side effects which unblind the study and amplify the placebo effect in the active treatment arm of the study. In the placebo arm of the study, the placebo effect may be decreased because patients are unblinded because they do not suffer from expected side effects. Unblinding due to side effects causes an unbalancing of the study and the principle of additivity, used to determine the pharmacological effect of the medicine, is invalid

Patients in a clinical trial are blinded to the treatment they are given because if they know they are taking the test medicine rather than a placebo, they may have an “expectancy” that the treatment will relieve their symptoms such as cough. Expectancy is a subjective sense of the probability of some future event, such as a belief that “my cough will get better because I am taking a new and effective cough medicine”. The role of expectancy on the placebo effect in clinical trial has been discussed in detail by experts in the field [13, 21]. It is important in clinical trials that participants’ belief about their treatment allocation is evenly distributed across treatment arms. If blinding is not maintained, for example due to side effects in the treatment arm, then expectancies are not adequately controlled and this causes a serious limitation to the validity of the trial [13]. “When blinding fails, the trial more closely resembles an open treatment versus no treatment comparison rather than the intended double-blind active treatment versus placebo comparison” [13]. The expectancy of patients who believe they are taking an active treatment will be greater than the expectancy of those patients who believe they are taking a placebo treatment, and this will unbalance the placebo effects of the two treatment groups as illustrated in Fig. 3. Despite the serious implications of unblinding in clinical trials, very few trials attempt to determine the success of blinding, and estimates indicate that blinding has failed in 23–60% of double-blind placebo-controlled trials and “failed blinding does make it impossible to rule out participant expectancy as the cause of the active treatment’s observed superiority over placebo” [13].

### How Unblinding Confounds a Clinical Trial

**Fig. 4:**
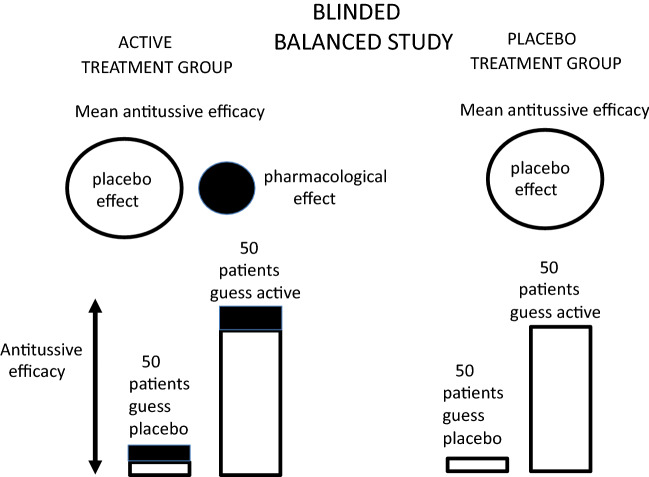
Magnitude of placebo effects in a properly blinded clinical trial. The magnitude of the antitussive efficacy is shown by the height of the column. The trial consists of 200 patients. 100 allocated to placebo and 100 allocated to an active medicine. The patients are asked at the end of the study to guess which treatment they were taking. Because the study is properly blinded in each arm of the study, the patient’s guesses as regards their treatment are no better than one would expect by chance with 50 patients in each of the four groups. This means that the mean antitussive efficacy for the two treatment groups is balanced

In a properly blinded clinical trial, the patient’s assumptions or guesses about which treatment they are taking are balanced as shown for a hypothetical clinical trial on 200 patients in Fig. 4. In the group of patients allocated to the placebo treatment arm (100), half the patients (50) believe they are taking a placebo and half (50) believe they are taking an active medicine, and the patient’s belief about their treatment is no better than one would expect by chance. Similarly, in the patients allocated to the active-medicine arm of the trial (100), half the patients (50) believe they are taking a placebo and half (50) believe they are taking an active medicine. For patients who believe they are taking a placebo treatment (100), there is little or no placebo effect, but for patients who believe they are taking an active treatment (100), there is a large placebo effect due to expectancy and belief about an active treatment. This hypothetical clinical trial is fully blinded, and the magnitude of the placebo effects in both treatment arms are balanced and any unbalancing of the study can be interpreted due to any pharmacological effect of the active treatment.

**Fig. 5:**
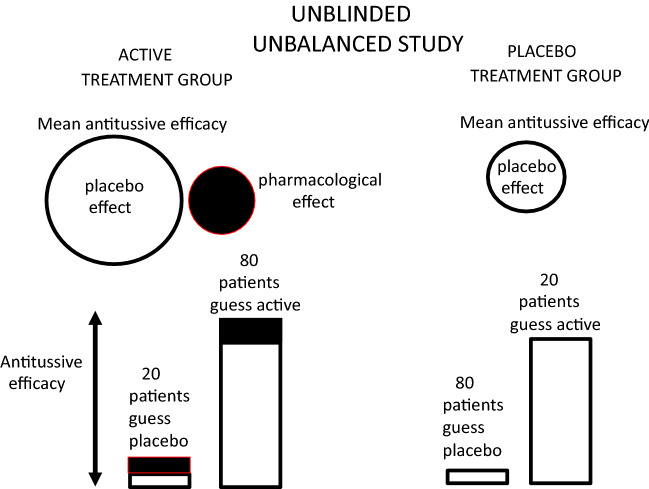
Magnitude of the placebo effects in a clinical trial where there is some unblinding of the treatments, perhaps due to incidence of side effects in the active medicine. The magnitude of the antitussive efficacy is shown by the height of the column. The trial consists of 200 patients. 100 allocated to take placebo and 100 allocated to take an active medicine. The patients are asked at the end of the study to guess which treatment they were taking. Because the study is partially unblinded, the patient’s guesses as regards their treatment are better than one would expect by chance. In the placebo treatment arm of 100 patients, 80 patients receiving placebo correctly guess their placebo treatment and only 20 patients receiving placebo incorrectly guess they are taking an active medicine. Similarly, in the active treatment arm, 80 patients receiving the active medicine correctly guess they are taking an active medicine and 20 patients incorrectly guess they are taking placebo. This means that the mean antitussive efficacy for the 100 patients in the active treatment arm is greater than the mean antitussive efficacy in the placebo treatment arm and this could be mis-interpreted as supporting the efficacy of the active medicine

In a hypothetical trial that is unblinded due to side effects of the active medicine, the patient’s assumptions or guesses about which treatment they are taking are unbalanced between the two treatment groups, and better than expected by chance alone, as shown in a trial on 200 patients in Fig. 5. In the group of patients allocated to the placebo treatment arm (100), a majority of the patients (80) correctly believe they are taking a placebo as they do not suffer from the expected side effects to the active medicine listed in the participant information, and a minority (20) believe they are taking an active medicine. Similarly, in the patients allocated to the active-medicine arm of the trial (100), the majority of patients (80) correctly guess they are taking an active medicine as they suffer from expected side effects associated with the active treatment, and only a minority (20) believe they are taking placebo. The combined placebo effect in the active control arm of the study is now much larger than the combined placebo effect in the placebo arm of the study due to the unblinding and the majority of patients correctly guessing their treatments. Because the magnitude of the placebo effects in both treatment arms is unbalanced, it is not possible to determine the magnitude of any pharmacological effect of the medicine. If the incidence of side effects was dose dependent, then this could be interpreted falsely as a dose-dependent effect of an active medicine, as the magnitude of the unbalancing of the study would increase with increase in the number of patients correctly guessing which treatment they were taking.

### Side Effects Confound Clinical Trials on New Cough Medicines

The search for new antitussive medicines has focussed recently on ATP antagonists that block the P2X2/3 ATP receptors on sensory nerves that mediate cough [10]. The problem with the development of ATP antagonists as antitussives is that ATP has a major role in the sensation of taste as first described in studies on rats [22] and later apparent in human studies on antitussives [10]. Clinical trials on the effects of ATP antagonists on chronic cough have all reported adverse effects on taste. One of the first antitussive studies on ATP receptor antagonism by Abdulqawi et al. (2015) reported that all of the patients receiving active treatment had taste disturbances (hypogeusia or dysgeusia), and that six patients discontinued the study due to taste disturbance [23]. All of the later studies on the antitussive efficacy of ATP receptor antagonists have reported side effects of the active treatment on taste (hypogeusia or dysgeusia), and the authors have expressed concerns that these side effects on taste may have unblinded the studies [9, 24, 25]. Unblinding of these studies does seem very likely because of the high incidence of taste-related adverse events which would be easily identified by the patients. In a recent study by Smith et al. (2020), 81% of patients taking a 50 mg dose of ATP antagonist reported a taste-related side effect [9]. The incidence of taste-related side effects was dose dependent (10% for 7.5 mg, 49% for 20.0 mg and 81% for 50.0 mg) with significant antitussive action of the ATP antagonist only found at the 50 mg dose which had the highest incidence of taste-related side effects [9]. The investigators acknowledged that the taste-related adverse effects of the active treatment had the potential to unblind the study [9].

The issue with these recent studies on new antitussives is that there was no attempt to determine the incidence of unblinding by questioning the patients at the end of the study as to which medication they believed they had been taking. If the level of unblinding followed the incidence of taste-related adverse effects, then potentially 81% of those patients on the highest dose of active treatment would have been unblinded, and this would invalidate the study by making it difficult or impossible to determine the efficacy of the active medicine above that of a placebo effect. The relationship between the dose of ATP antagonist and the incidence of side effects could also lead to a false assumption about a dose-dependent pharmacological effect of the active medicine as the dose dependency would relate to a dose-dependent placebo effect rather than a dose-dependent pharmacological effect on cough.

### Is there Any Way to Improve Clinical Trials on New Antitussives?

Unblinding of clinical trials on antitussives is a major issue in trials on ATP antagonists because of their readily recognisable side effects on taste. It may be possible to develop ATP antagonists that do not have major side effects on taste, and this is an ongoing project with new selective ATP antagonists [26]; however, side effects are likely to be an issue with new antitussive clinical trials and the following discussion looks at ways that their confounding effects may be mitigated.

### Participant Information

The participant information about side effects of medicines in clinical trials is an important part of the clinical trial protocol and there are serious ethical and legal issues about not providing full information about potential side effects. However, when side effects are described in the participant information, patients taking the active treatment will be unblinded when they suffer from the expected side effects and patients taking placebo may correctly guess that they are taking a placebo when they do not suffer from the expected side effects. The other issue about describing side effects is that this may increase the nocebo effect and cause increased reporting and severity of side effects, not only in those taking active treatments but also in those taking a placebo treatment [27–29].

It is self-evident that not all patients in a clinical trial are the same and that they will respond differently to warnings about side effects, and it has been proposed that participant information can cause harm to patients, and that information should be more tailored to the differences between patients as regards the way they cope with information on side effects [30]. This approach seems to be unrealistic, as several versions of the participant information would be needed and it would entail questioning and screening of patients to determine which version of the participant information they should receive.

An important aspect of the participant information and informed consent is to inform the patient about possible risks in participating in a clinical trial and this is where it may be possible to overcome some of the confounding effects of side effects unblinding a trial. The patient could be informed about serious side effects of treatment that may cause permanent harm such as risk to life, disability or disturbing side effects such as bleeding but would not be informed about side effects that would be expected to disappear on discontinuation of the test medicine, such as effects on taste as seen in the trials on ATP antagonists. A statement could be made in the participant information that other side effects may occur with the treatment, but these would only occur during the treatment period and would not put the patient at risk. This would protect the investigator against litigation for serious harm to patients but would help to blind the study. However, in the age of Google searches, patients may still easily discover the range of side effects associated with test medicines, and this could unblind studies. There are also the ethical and legal issues in not providing a full description of possible side effects, and in the present framework of ethical approval of clinical trials, it seems unlikely that investigators would be allowed to provide incomplete information about possible side effects. Another way in which the participant information could be used to reduce unblinding is by informing participants that drug side effects are commonly associated with those taking placebo treatment [31] and that the presence of a side effect does not necessarily indicate that they are taking an active medicine.

### Active Placebos

If a test medicine in a clinical trial has a side effect such as “dry mouth”, then it may be possible to maintain blinding of the trial by a placebo treatment that also causes dry mouth, an “active placebo”. A recent review on the use of active placebos in clinical trials reported that they are rarely used, and the main argument for their use was to reduce the risk of unblinding, whereas the main argument against their use was the risk of an unintended therapeutic effect [32]. Atropine has been used as an active placebo in clinical trials on psychoactive medicines that have the side effect of dry mouth, but a major issue has been that the psychoactive medicines have low efficacy, and the active placebo has performed much better than expected, leading investigators to propose that atropine could have significant psychoactive efficacy [6]. Similarly, a study on the use of atropine as an active placebo in a trial involving the analgesic diclofenac led the investigators to conclude that atropine could have significant analgesic efficacy [20]. These unintended therapeutic effects of atropine and other agents used as active placebos are not the only issues in the use of active placebos, as there is also an ethical issue about causing unnecessary side effects and possible harm to those on placebo treatments.

When side effects are clearly recognised such as those on taste in trials on antitussive ATP antagonists, it is difficult to see how an active placebo such as atropine could help in blinding a study. The use of any other medicine as an active placebo that may affect taste causes complications in ethical approval of the study and potentially unexpected therapeutic effects.

### Cross-Over Studies

The use of double-blind placebo-controlled cross-over studies in clinical trials on antitussives has been previously discussed [33]. A cross-over design may be useful in assessing a medicine that is only marginally more effective than placebo, as the cross-over amplifies the efficacy of the active arm of the study by enhancing the placebo effect and decreasing the placebo effect in the placebo arm of the study. In this regard, a crossover study unbalances and unblinds the study. However, this design will also perform in the same way for medicines with side effects by amplifying and unblinding the active arm of the study when patients detect the side effects. Cross-over studies on psychoactive medicines have reported that readily recognisable side effects lead to unblinding of the study and overestimation of the benefits of the test medicine [34]. A cross-over design is therefore likely to give a false indication of efficacy in the case of medicines with significant side effects.

### Patient Selection

There is variability in the magnitude of the placebo effect in patients, and some patients are referred to as “placebo responders” and others as “non responders”, and it has been proposed that clinical trials could eliminate placebo responders in order to favour the active treatment group [35]. However, a study on psychoactive clinical trials found that “There was no statistically significant difference in effect size between the clinical trials that had a placebo run-in phase followed by withdrawal of placebo responders, and those trials that did not [36]”.

Cough is under voluntary control [2] and the magnitude of the placebo response may be related to the ability of patients to suppress cough. A study on patients with acute cough has shown that the ability of patients to control cough is correlated to the severity of cough [37]. Studies on new cough medicines have reported that “Patients with the highest baseline cough frequency experienced the greatest improvements” [25], and this may be related to the ability of patients to control their coughing. It would therefore seem reasonable for trials to recruit patients with high levels of cough severity as these patients are less likely to be able to control cough and may have a less tendency to respond to placebo treatments. However, a complicating factor is that recruiting only patients with severe cough measurements may mean that some of the perceived efficacy of a treatment is due to the measure regressing towards a mean value [38].

### Assessment of Blinding

The blinding of patients and all those concerned in the conduct of clinical trials is a fundamental aim in double-blind clinical trials, but it receives less attention than other important components of the trial, such as randomisation or compliance [39]. More than 90% of publications of blinded trials do not report on the risk of unblinding [40] and a search of the literature has not found any clinical trials on cough medicines that have assessed the blinding of the study.

The blinding of a study may be assessed early after starting treatment or more often on completion of the study by asking patients which medication they think they have received. Some patients may respond that they do not know their allocation and this data can also be collected and assessed [39]. The degree of blinding can be quantified as a blinding index and various measures have been proposed, as well as how to calculate a sample size for blinding assessment [41–43].

Unblinding may not invalidate the results of a clinical trial as patients may be unblinded not only because they sense side effects associated with treatment but also because they appreciate that the treatment is effective and is “working”. With clinical trials that are conducted over a period of weeks, such as those on chronic cough treatments, it may be possible to separate unblinding due to efficacy, and unblinding due to side effects, if the pharmacodynamics of these two effects are different. If side effects occur soon after treatment but significant efficacy only occurs after weeks of treatment, then this may indicate that the unblinding due to early recognition of side effects is not a major issue in the perceived efficacy of the cough medicine.

A good example of how assessment of blinding may be used to interpret the results of a trial that was partially unblinded is discussed by Moscucci et al. (1987) and these authors acknowledge that it is difficult to maintain complete double-blinding in trials, and that is why it is important to assess the degree of blinding, and that the results of trials that failed to achieve complete blinding should not be discarded [12].

## Discussion

In recent clinical trials on antitussives, side effects of the medicines are acknowledged as possibly unblinding the studies but there is no discussion on how this may impact on the validity of the studies. There is also no attempt to try and quantify the scale of the problem of unblinding by questioning patients. It appears that the problem of unblinding is acknowledged but then discounted as of no real consequence because this issue needs to be neutralised. However, without any measure of unblinding in the studies, it is not possible to assess the validity of the studies and determine the real pharmacological benefit of new antitussives. The issue of unblinding of clinical trials is not a new issue but if real progress is to be made in developing new antitussives, then it is an issue that needs to be urgently addressed.
